# Impact of telemonitoring home care patients with heart failure or chronic lung disease from primary care on healthcare resource use (the TELBIL study randomised controlled trial)

**DOI:** 10.1186/1472-6963-13-118

**Published:** 2013-03-28

**Authors:** Iñaki Martín-Lesende, Estibalitz Orruño, Amaia Bilbao, Itziar Vergara, Mª Carmen Cairo, Juan Carlos Bayón, Eva Reviriego, María Isabel Romo, Jesús Larrañaga, José Asua, Roberto Abad, Elizabete Recalde

**Affiliations:** 1 Bilbao Primary Care Health Region, Osakidetza – Basque Health Service, Bizkaia, Spain; 2 Basque Office for Health Technology Assessment (OSTEBA), Department of Health and Consumer Affairs, Basque Government, Vitoria-Gasteiz, Araba, Spain; 3 Research Unit, Basurto University Hospital, Osakidetza, Health Services Research on Chronic Patients Network (REDISSEC), Bilbao, Bizkaia, Spain; 4 Primary Care Research Unit of Gipuzkoa, Osakidetza, REDISSEC, Donostia, Gipuzkoa, Spain

**Keywords:** Telemonitoring, Primary care, Effectiveness, Hospital admissions, In-home patients, Chronic diseases, Heart failure, Chronic lung disease, Elderly

## Abstract

**Background:**

There is growing evidence that home telemonitoring can be advantageous in societies with increasing prevalence of chronic diseases.

The main objective of this study is to evaluate the effect of a primary care-based telemonitoring intervention on the number and length of hospital admissions.

**Methods:**

A randomised controlled trial was carried out across 20 health centres in Bilbao (Basque Country, Spain) to assess the impact of home telemonitoring on in-home chronic patients compared with standard care. The study lasted for one year. Fifty-eight in-home patients, diagnosed with heart failure (HF) and/or chronic lung disease (CLD), aged 14 or above and with two or more hospital admissions in the previous year were recruited. The intervention consisted of daily patient self-measurements of respiratory-rate, heart-rate, blood pressure, oxygen saturation, weight, body temperature and the completion of a health status questionnaire using PDAs. Alerts were generated when pre-established thresholds were crossed. The control group (CG) received usual care. The primary outcome measure was the number of hospital admissions that occurred at 12 months post-randomisation. The impact of telemonitoring on the length of hospital stay, use of other healthcare resources and mortality was also explored.

**Results:**

The intervention group (IG) included 28 patients and the CG 30. Patient baseline characteristics were similar in both groups. Of the 21 intervention patients followed-up for a year, 12 had some admissions (57.1%), compared to 19 of 22 controls (86.4%), being the difference statistically significant (p = 0.033, RR 0.66; 95%CI 0.44 to 0.99). The mean hospital stay was overall 9 days (SD 4.3) in the IG versus 10.7 (SD 11.2) among controls, and for cause-specific admissions 9 (SD 4.5) vs. 11.2 (SD 11.8) days, both without statistical significance (p = 0.891 and 0.927, respectively). Four patients need to be telemonitored for a year to prevent one admission (NNT). There were more telephone contacts in the IG than in the CG (22.6 -SD 16.1- vs. 8.6 -SD 7.2-, p = 0.001), but fewer home nursing visits (15.3 -SD 11.6- vs. 25.4 -SD 26.3-, respectively), though the difference was not statistically significant (p = 0.3603).

**Conclusions:**

This study shows that telemonitoring of in-home patients with HF and/or CLD notably increases the percentage of patients with no hospital admissions and indicates a trend to reduce total and cause-specific hospitalisations and hospital stay. Home telemonitoring can constitute a beneficial alternative mode of healthcare provision for medically unstable elderly patients.

**Trial registration:**

Current Controlled Trials ISRCTN89041993

## Background

Home telemonitoring comprises the use of information and communication technology from the patient home so that clinical parameters and other clinical data can be sent, both digitally or over the telephone, to the health professionals managing the patient care [1]. The regular collection of such medical information allows professionals or clinical support teams conducting comprehensive monitoring of patients with chronic and complex conditions and adjusting treatments, as well as facilitating the early identification of worsening episodes, which if not detected quickly often lead to emergency department attendances and/or hospital admissions [1,2]. As demonstrated by various studies, such as the Strategic Intelligence Monitor on Personal Health Systems phase 2 (SIMPHS 2) in Europe, this is an area that is undergoing exponential growth with the technology being ever more widely deployed [1,2]. This tendency can be attributed to health systems seeking new approaches and strategies to respond to a growing demand for health and social resources due to population ageing and the associated growth in the prevalence of chronic disease and comorbidity [1].

Recently, a number of literature reviews have been published providing new evidence on the usefulness of home telemonitoring in the management of patients with chronic diseases [3-6]. Our team commissioned a systematic literature review to assess the effectiveness of home telemonitoring in heart failure (HF) and chronic obstructive pulmonary disease (COPD) [3]; 12 reviews and 24 randomised controlled trials (RCTs) on HF and 3 reviews and 7 RCTs on COPD were analysed. In patients with HF, telemonitoring showed a reduction in mortality and in the number of all-cause hospitalisations, as well as a positive effect on quality of life and on adherence to treatment; while there was not a clear trend in the length of hospital stay, compared to usual care. In patients with COPD, both telemonitoring and structured telephone support reduced the number of all-cause hospital admissions; but there were no conclusive data on mortality or use of other healthcare resources [3].

Further, two systematic literature reviews on home telemonitoring have so far been published by the Cochrane Collaboration. Initially, in 2000, the effects of telemedicine were assessed compared to face-to-face care, showing that there was little evidence on the clinical benefits of telemedicine and that new research was needed to explore the potential benefits of the new technology [7]. More recently, another Cochrane review assessed the effect of structured telephone support and telemonitoring programmes compared to standard care in patients with HF, concluding that both approaches had the effect of reducing the risk of all-cause mortality and hospital admissions in these patients, as well as improving quality of life (QoL) and decreasing healthcare costs [4].

Additionally, two other recent systematic reviews and meta-analyses have been undertaken in order to assess the effect of telemonitoring in patients with HF [5] and COPD [6]. In HF patients, telemonitoring was shown to reduce mortality, and several studies suggested that hospitalisations and healthcare resources use were also decreased. In COPD patients, the authors found that both telephone contact and device-based telemonitoring reduced hospital admissions and emergency department attendances [5,6].

In the United Kingdom, the Whole System Demonstrator (WSD) [8,9], the largest RCT to date in this field, including 3,230 patients and 179 general practices was launched in 2008. The study assesses the impact on the use of healthcare services and quality of life of a two-component intervention: telecare (remote monitoring of individuals’ lifestyle and safety confirmation) and telehealth (which involves the telemonitoring of 3,230 patients with diabetes, HF or COPD). The results of this study will provide a solid evidence base. Some preliminary findings have already emerged and suggest reductions in hospital admissions and length of stay, emergency department visits, mortality and costs [9].

HF and chronic lung disease (CLD) (witch mostly comprises COPD and asthma) are both characterised by the functional limitation and clinical progression. In fact, the prospect of new readmissions and patient survival are generally worsened by the hospital admissions derived from these conditions. Yet, the quality of the care for patients affected by these diseases can be improved by changing from a model based on the management of destabilisation within hospitals to a model focused on the maintenance of the basal status (through structured telephone support or telemonitoring in the home) [3]. Nevertheless, to date, there is a paucity of research with the focus on interventions in which the management of the telemonitoring systems lays directly into the hands of primary care professionals (at local health centres). Moreover, few studies have considered the monitoring of more than one disease through the same telemonitoring system and, as a result, there are more data available for some diseases than others, the most consistent findings having been obtained for HF [10]. The present study addresses the gaps in the existing literature and highlights the importance of primary care in the management of chronic patients and the recognition of comorbidity as one of the main characteristics of such patients [11].

This paper reports the results of a randomised controlled trial, the TELBIL study. The trial protocol has been published elsewhere [12]. The main objective of the study was to assess the effect of a primary care-based telemonitoring system on the number and length of hospital admissions in patients with HF and/or CLD at 12 months post-randomisation compared with the standard health care practice. In addition to the effect on hospitalisations, we also report the impact of the telemonitoring intervention on the use of other healthcare resources (emergency department attendances, home visits by primary care professionals, appointments at the health centre or with specialists, and telephone calls), and on mortality, as well as the association between hospitalisations and alerts generated by the telemonitoring system in the five days prior to the hospital admissions.

## Methods

### Study design and setting

**Figure 1:**
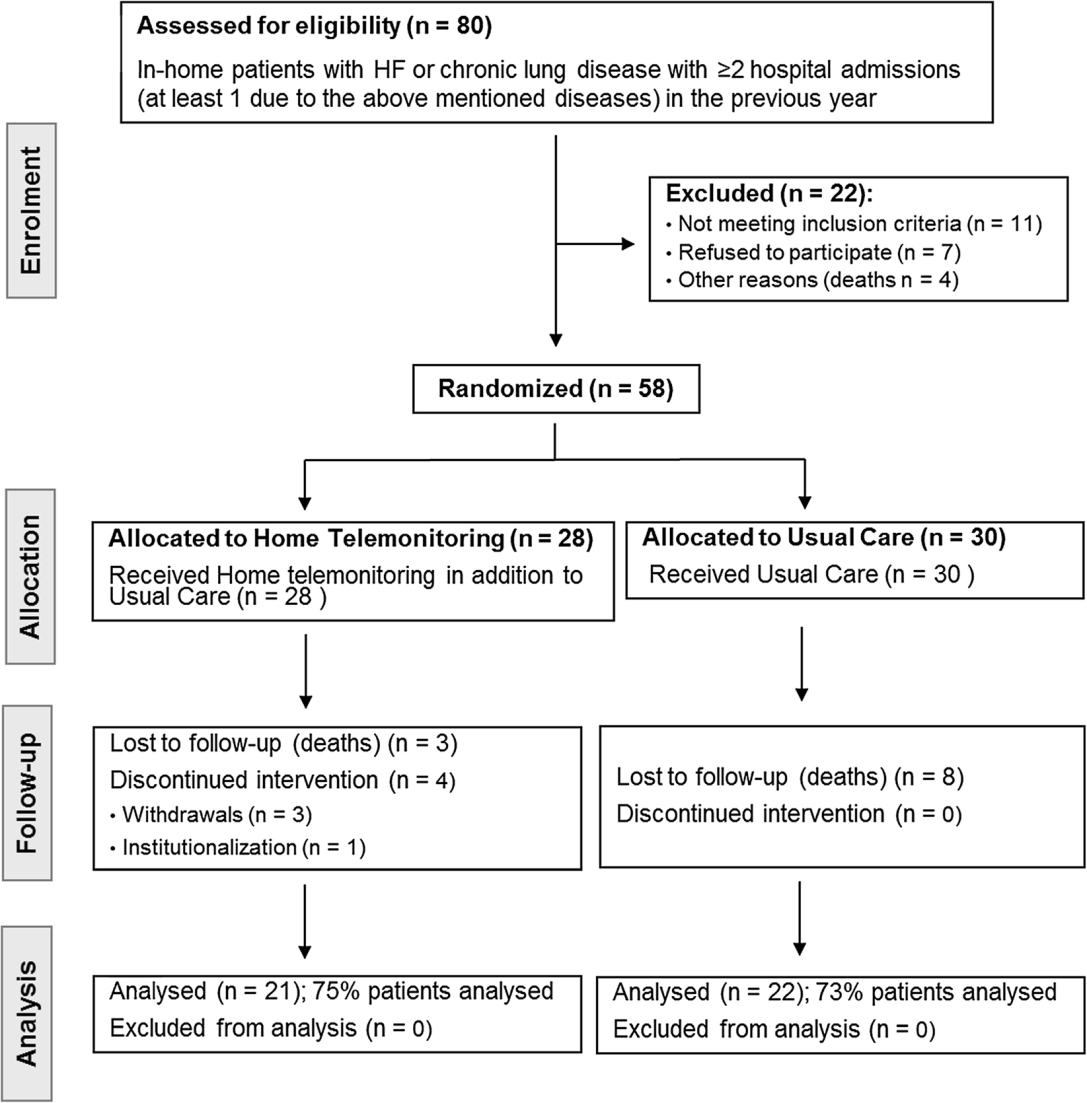
Flow of participants through the TELBIL trial.

This is a randomised controlled trial with a one-year follow-up and analysis at 3, 6 and 12 months post-randomisation. In the intervention group (IG), in addition to the standard practice, patients were monitored using a telemonitoring procedure, while the control group (CG) received usual care. The intervention ran from February 2010 to August 2011, including the period of patient recruitment over the first 6 months, with the participation of 20 of the 24 health centres in the Bilbao Primary Care Health District. This urban health region serves a catchment population of 390.000 people, of whom around 27% are over 60 years old. The team of professionals in charge of the first level of healthcare is made up of 239 general practitioners (GPs), 326 nurses and 40 pediatricians. Acceptance to participate was an inclusion criterion for both health professionals and patients. Figure 1 outlines the general study design, showing the flow of patients through the trial. Further details concerning the methodology of the trial can be found in the published study protocol [12].

### Eligible patients

Home care adult patients (individuals who receive routine healthcare at home due to lack of or severe difficulties with mobility), diagnosed with HF and/or CLD, aged 14 or above, with a history of at least two hospital admissions in the previous year, at least one of these admissions having been associated with one of the two conditions under study were included in the trial. The patients excluded from the study were the following: those in residential care, patients receiving regular monitoring or treatment by specialists or hospital services (such as, patients on hemodialysis or rehabilitation programmes), those in the terminal phases (with a life expectancy under 6 months) of other illnesses (not HF or CLD) patients with established cognitive impairment and those not willing to participate were excluded from the study. Patient randomisation and recruitment processes are described in detail in the study protocol [12].

### Ethics approval

The study was approved by the Ethics Committee for Scientific Research (CEIC, Basurto University Hospital, Bizkaia). Patients or relatives gave written informed consent prior to participating in the study. Patients’ identity was preserved at all times during the course of the trial.

### Description of the intervention

In addition to the usual care, the IG was followed-up through by telemonitoring, which consisted of daily transmissions from the patients’ homes of the following self-measured clinical parameters using a smart phone-personal digital assistant (PDA) with the help of the caregivers: blood oxygen saturation, blood pressure, heart and respiratory rates, body weight and temperature; the first three parameters being transferred to the PDA by Bluetooth wireless technology. Additionally, patients completed a brief health status questionnaire aimed at assessing the patient’s perception of his/her medical and functional condition. The questionnaire also contained items concerning compliance with medication and diet. All data were stored on a Web-based platform and were accessible by health professionals (*i.e.*: the patient’s assigned general practitioner (GP) and nurse at the health centre) during business hours from Monday to Friday. The telemonitoring system comprised personalised alerts set for each patient, with messages being sent to the Web platform when the recorded parameters fell outside the pre-established limits (which could be adjusted over time). The established threshold values were essential for the monitoring of the patient’s condition and for the detection of unusual changes. When the measurements fell outside the established limits, alerts were triggered via the PDA terminal and the clinical staff acted according to the medical condition of the patient. Real alerts were differentiated from false alarms through the assessment of all the clinical information and the health status questionnaire received through the telemonitoring system. Therefore, even if an alarm was triggered for a specific clinical parameter, it was the patient’s overall health status that was taken into consideration by healthcare professionals, before taking any further action. Patients were advised to call the emergency services through the telemonitoring system at weekends and at times when the health centres were closed.

In the CG, patients received only usual care, consisting of regular medical examinations in line with the established programmes for monitoring home-based patients. The frequency of the medical examinations varied depending on the clinical, social and family situation of each patient. Additionally, the GP and/or nurse visited or called the patient on demand in the event of a deterioration in the medical condition.

All health professionals participating in the study (for both, IG and CG) received specific training aimed at strengthening and standardising the management of the clinical conditions under study.

### Study variables

#### Initial assessment and baseline data

The variables described below were analysed. At baseline, (i.e., at the moment when patients were included in the study), sociodemographic and clinical data were collected together with the use of healthcare resources over the previous year:

Patient’s *sociodemographic data* collected included: date of birth and age, gender, health centre, assigned GP and nurse, level of education, social and family characteristics (aiming at identifying a lack of support on the basis of assessing factors including poverty, loneliness, isolation, social exclusion and recent widowhood).

The following *clinical data* were also recorded: diagnosis of HF or CLD, based on the computerised primary care registry and hospital medical records, specifying the aetiology, degree of severity of the disease (based on the FEV1 – forced expiratory volume in one second – for COPD and on the NYHA – New York Heart Association – classification and the ejection fraction for HF), requirements of home oxygen therapy; the Charlson comorbidity index score [13]; regular medication (taken from medical records and confirmed by health professionals and/or patients themselves or their relatives); treatment adherence measured using the Morisky Adherence Scale (the original 4-item version) [14]; the number of hospital admissions (noting whether they were cause-specific, that is, related to the conditions under study) and the mean duration of the hospital stay; and the use of other healthcare resources during the year prior to the inclusion in the study (emergency department attendances; appointments with specialists; home visits, including both scheduled visits and those prompted by the telemonitoring of the patient; and other contacts with health centre professionals by telephone or face-to-face, even if patients themselves did not attend, for administrative tasks, follow-up, prescriptions, etc.).

#### Outcome measures

The primary outcome measure was the number of hospital admissions that occurred in a period of 12 months post-randomisation. The hospitalisations were classified as due to HF, due to CLD (i.e., COPD, asthma and other respiratory conditions) and non-cardiorespiratory causes. Secondary outcome measures included the length of hospital stay (number of bed-days for emergency admissions with a primary diagnosis of HF, CLD and other causes during 12 months after randomisation), mortality rates and use of other healthcare resources (emergency department attendances, home visits, appointments at the health centres and with the specialists and telephone calls). We also assessed the relationship between the hospitalisations and the number of alerts generated by the telemonitoring system in the five days leading up to the corresponding admissions.

### Sample size

Prior to the start of the trial, we had estimated that thirty patients would be recruited in each of the study groups, the main constraints being the number of available devices and the number of potential patients fulfilling the inclusion criteria in the Bilbao Primary Care Health Region. Thus, if 30 patients had been included in each group, and assuming a 10% loss to follow-up at 12 months, we estimated a statistical power of 72% to detect significant differences between the CG and the IG in the mean number of total admissions with a level of significance of 5% (assuming that the mean number of admissions in the CG had been 3.5, the standard deviation 1.7, and there had been a 35% decrease of hospitalisations in the IG with respect to the CG). However, there have been some variations with respect to the statistical power on completion of the trial due to recruitment limitations and follow-up losses, which will be explained in detail in the discussion section.

### Statistical analysis

For the descriptive analysis, frequencies and percentages were calculated for categorical variables, and mean and standard deviation (SD) or median and interquartile range (IQR) for continuous variables. Baseline sociodemographic and clinical data of the two groups (IG and CG) were compared to assess the homogeneity. Both the primary and secondary outcome measures (number of hospital admissions and length of hospital stay, mortality rates, and use of healthcare resources) were also compared between the two groups of patients at 3, 6 and 12 months. For the comparison of qualitative variables Chi-square or Fisher’s exact tests were used, while for quantitative variables Student’s t-tests or non-parametric Wilcoxon tests were employed, respectively, depending on whether or not the data were normally distributed.

For the number of hospital admissions at 12 months post-randomisation, the primary outcome measure, we estimated the relative risk (RR) of the occurrence of at least one admission (for any cause) in intervention patients compared to control patients. Additionally, the incidence rate ratios for total and cause-specific admissions in intervention patients compared to controls was also estimated. Finally, we compared the risk of admissions for all causes and for causes related to the conditions under study at 12 months of follow-up, in the two groups of patients after adjusting for the baseline variables found to be statistically significant in the assessment of group homogeneity. For this purpose, logistic regression analysis was applied, considering the occurrence of at least one admission as the dependent variable, the assigned group (CG vs. IG, the main independent variable) and other adjustment variables (i.e., lack of social support) as independent variables. The data are presented as odd ratios (OR) with 95% confidence intervals (95% CI). In addition, we estimated the number needed to treat (NNT), in terms of provision of telemonitoring support to prevent one hospital admission in a year. For the calculation of the NNT the relative risk of having no hospital admission in the IG and in the CG was taken into consideration (NNT = 1/proportion of patients with no admissions in the IG – proportion of patients with no admissions in the CG), and its 95% CI [15].

On the other hand, we compared the variables describing attendances and use of other healthcare resources in the year prior to the inclusion in the study and after 12 months of follow-up in each group of patients, using the paired t-test or the non-parametric Wilcoxon signed-rank test when the normality assumption was not met. Furthermore, differences in the changes in these variables between the two groups of patients (IG and CG) were assessed, using the Student’s t-tests or non-parametric Wilcoxon tests.

The level of statistical significance was set at p < 0.05. The analyses were performed using SAS for Windows, version 9.2 (SAS Institute, Cary, NC, USA) and the IBM SPSS Statistics, version 19 (IBM, Somers, NY, USA).

## Results

We recruited and randomly allocated 28 patients (from 14 different health centres) to the intervention group and 30 patients (from 6 different health centres) to the control group. Twenty-two candidate patients were excluded before randomisation: 11 did not meet the inclusion criteria (4 were institutionalised, 4 were not home care patients and 3 were difficult to follow-up), 5 declined to participate and 2 whose corresponding health professionals did not wish to take part in the study, while the other 4 died. The 12-month follow-up was completed by 21 patients in the IG (of the initial 28 patients, 3 died, 3 withdrew from the study and 1 was institutionalised), and by 22 patients in the CG (of the initial 30 patients, 8 died during the study period).

### Baseline characteristics of the participating patients

The mean age of the 58 patients included in the study was 81 (SD 7.5) years, and 58.6% of them were men. Overall, 46.5% of patients had the two medical conditions targeted in the trial (HF and CLD) simultaneously, while 27.6% had only HF and 25.9% had only CPD. More than half (57.1%) needed permanent oxygen therapy in the home, 86.2% had a Charlson index score above 2 (high comorbidity), and only 12% obtained a Barthel Index score above 90 (which indicated little dependence) as opposed to 34.5% of patients scoring below 60 (showing severe or total dependence). The patients included in the study took an average of 10.6 (SD 3.2) medications every day and during the previous year to the start of the study were visited at home a median of 22.5 times (range 3 to 139) and had a median of 3 hospitalisations (range 2 to 9). At baseline, the above mentioned characteristics were similar for the patients in the two groups across all the variables except that those in the IG had a significantly lower level of social support (p = 0.038), as shown in Table 1. With regard to the specific diagnoses within the medical conditions under study, the most common cause of HF was ischaemic heart disease (34.9%), and 78.6% of patients with CLD had COPD with different levels of severity: moderate (17.4%), severe (21.7%) and very severe (60.9%). No statistically significant differences were observed in the diagnoses between the study groups (p = 0.723). In the period prior to the study, home visits by the health professionals were carried out by nurses in 77.3% of cases, and there were no statistically significant differences in the number of nurse home visits between the two groups, with a median of 11.5 (IQR 7.5 to 23.5) visits for the patients in the IG and a median of 21 (IQR 9 to 28) for the patients in the CG (p = 0.169); similarly the number of home visits conducted by the GPs were comparable, with a median of 6 (IQR 3 to 7) and 4.5 (IQR 2 to 8) visits in the intervention and control groups, respectively (p = 0.303).

**Table 1:** Comparison of the baseline sociodemographic characteristics, clinical characteristics and use of healthcare resources in the intervention and control groups

	**IG**	**CG**	**p-value **^**I**^
	**(n = 28)**	**(n = 30)**	
**Sociodemographic variables**			
Sex, n (%)			0.198
Men	14 (50%)	20 (66.7%)	
Women	14 (50%)	10 (33.3%)	
Age (years), mean (SD)	80.7 (9)	81.3 (6)	0.653
Living with:, n (%)			0.553
alone	4 (14.3%)	2 (6.7%)	
spouse/partner	11 (39.3%)	15 (50%)	
Others (other relatives, formal career, etc.)	13 (46.4%)	13 (43.3%)	
Caregiver, n (%)			0.149
Spouse/partner	10 (35.7%)	16 (53.3%)	
Daughter	9 (32.1%)	8 (26.7%)	
Other relative	4 (14.3%)	0 (0%)	
Other	5 (17.9%)	6 (20%)	
Lack of social support ^II^, n (%)	8 (28.6%)	2 (6.7%)	0.038
**Clinical and functional variables**			
Disease-related reason for inclusion, n (%)			0.596
Heart failure	6 (21.2%)	10 (33.3%)	
Lung disease	8 (28.6%)	7 (23.3%)	
Both	14 (50%)	13 (43.4%)	
Home oxygen therapy, n (%)	16 (57.1%)	14 (46.7%)	0.425
Comorbidity Charlson Index ≥2, n (%)	24 (85.7%)	26 (86.7%)	1
Indicators of clinical deterioration ^III^, n (%)	21 (75%)	25 (83.3%)	0.434
Conditions to determine frequent use of healthcare services ^IV^, n (%)	19 (67.9%)	24 (80%)	0.291
Adequate treatment adherence (Morisky Adherence Scale), n (%)	28 (100%)	29 (96.7%)	1
**Use of healthcare resources** over the year prior to inclusion			
Regular medicines/day, mean (SD)	10.1 (3.1)	11.1 (3.3)	0.240
All-cause hospitalisations, mean (SD)	3.4 (1.7)	3.4 (1.7)	0.981
Cause-specific hospitalisations^V^, mean (SD)	2.6 (1.5)	2.6 (1.5)	0.891
Length of stay (days/admission), mean (SD)	11.3 (6)	10.4 (7)	0.207
Emergency department attendances not resulting in admission, median (IQR)	1 (0 – 5)	1 (0 – 6)	0.920
Appointments with specialists, median (IQR)	3 (0 – 13)	2.5 (0 – 12)	0.129
Home visits, median (IQR)	20.0 (3 – 139)	23.5 (3 – 67)	0.291
Telephone contacts, median (IQR)	3 (0 – 22)	3.5 (0 – 20)	0.619

IG: intervention group; CG: control group. The data are expressed as frequency and percentage (%) for categorical variables, and as mean with the standard deviation (SD) or median with the interquartile range (IQR) for continuous variables.

^I^ Chi-square or Fisher’s exact tests were used to compare categorical variables and Student’s t-tests or non-parametric Wilcoxon tests to compare continuous variables.

^II^ Based on the assessment of social factors including poverty, loneliness, isolation, social exclusion, and recent widowhood.

^III^ Presence of at least one of the following characteristics: bone and joint disease, ischaemic heart disease, sequelae of cerebrovascular accident, Parkinson’s disease, diabetes, obesity (body mass index >30), visual or hearing impairment, current mental illness (receiving treatment), or other factors considered relevant by the patient’s doctor and documented.

^IV^ On acenocoumarol and/or presence of pressure ulcers and/or need for regular dressing changes or injections.

^V^ Admissions to hospital for respiratory and heart-related problems.

### Effect of home telemonitoring on hospital admissions

At 12 months of follow-up, the RR of hospitalisation for the patients in the IG compared to those in the CG was 0.66 for all-cause hospital admissions (95% CI 0.44 to 0.99, p = 0.033) and 0.74 (95% CI 0.48 to 1.14, p = 0.159) when only the cause-specific admissions (for respiratory or heart-related problems) were considered (see Table 2). Of the 21 patients in the IG who completed the 12-month follow-up, 9 had no hospital admissions at all, compared to 3 among the patients in the CG (p = 0.033) (with 9 and 5 patients with no admissions in the IG and CG respectively if cause-specific hospitalisations were considered). In patients who completed the 12-month follow-up, the NNT with telemonitoring to prevent one hospitalisation over a year was 4 for all-cause admissions (95% CI 2 to 28) and 5 for cause-specific admissions, the latter not being statistically significant. Considering the total number of days of follow-up of all the patients who initiated the study (8,828 and 9,970 in the IG and the CG, respectively), all-cause admissions (53 and 68 in the IG and CG, respectively) and cause-specific admissions (44 and 60 in the IG and the CG, respectively), the incidence rate ratios between the patients in the IG and CG were 0.88 (95% CI 0.61 to 1.26) for all-cause admissions and 0.83 (95% CI 0.56 to 1.22) for cause-specific admissions. These correspond to 12% and 17% lower rates of hospitalisations among patients in the IG as compared to patients in the CG. Further, the OR of all-cause admission at 12 months post-randomisation for patients in the IG compared to those in the CG was 0.21 (95% CI 0.05 to 0.94, p = 0.04). Logistic multivariate regression analysis was conducted, showing that the independent variable (i.e., lack of social support) was non-significant. Thus, the aforementioned multivariate model was not considered for subsequent analysis.

**Table 2:** Hospital admissions and length of stay during the follow-up period in the intervention and control groups

	**At 3 months**			**At 6 months**			**At 12 months**		
	**IG**	**CG**	**p**	**IG**	**CG**	**p value**^**I**^	**IG**	**CG**	**p value**^**I**^
	**(n = 25)**	**(n = 29)**	**value**^**I**^	**(n = 25)**	**(n = 28)**		**(n = 21)**	**(n = 22)**	
**All-cause hospitalisations**			0.744			0.887			0.250
mean (SD)	0.5 (0.8)	0.4 (0.6)		1.2 (1.7)	1 (1.3)		2.1 (2.8)	2.1 (1.5)	
median (IQR)	0 (0–1)	0 (0–1)		0 (0–2)	1 (0–1)		1 (0–3)	2 (1–3)	
**All-cause hospitalisations categorised**			0.907			0.506			0.033
0 admisssions, n (%)	16 (64%)	19 (65.5%)		13 (52%)	12(42.9%)		9 (42.9%)	3 (13.6%)	
≥1 admisssions, n (%)	9 (36%)	10 (34.5%)		12 (48%)	16 (57.1%)		12 (57.1%)	19 (86.4%)	
RR (95% CI)	1 (0.5 – 2.2)			0.8 (0.5 – 1.4)			0.7 (0.4 – 0.9)		
**Cause-specific hospitalisations**^II^			0.895			0.732			0.328
mean (SD)	0.4 (0.7)	0.3 (0.6)		0.9 (1.3)	0.9 (1.3)		1.8 (2.6)	1.8 (1.6)	
median (IQR)	0 (0–1)	0 (0–1)		0 (0–1)	0.5 (0–1)		1 (0–2)	1.5 (1–2)	
**Cause-specific hospitalisations categorised**			0.973			0.465			0.159
0 admisssions, n (%)	18 (72%)	21 (72.4%)		15 (60%)	14 (50%)		9 (42.9%)	5 (22.7%)	
≥1 admisssions, n (%)	7 (28%)	8 (27.6%)		10 (40%)	14 (50%)		12 (57.1%)	17 (77.3%)	
RR (95% CI)	1 (0.4 – 2.4)			0.8 (0.4 – 1.5)			0.7 (0.5 – 1.1)		
**Length of stay (days/admission)**^III^									
all-cause hospitalisations, mean (SD)	8.7 (2.3)	11.2 (8.9)	0.954	8.2 (3.3)	8.2 (5.3)	0.477	9.0 (4.3)	10.7 (11.2)	0.891
cause-specific hospitalisations, mean (SD)	8.9 (2.4)	11.6 (9.1)	0.936	8.4 (3.2)	8.3 (5.0)	0.497	9.0 (4.5)	11.2 (11.8)	0.927

IG: intervention group; CG: control group; RR: relative risk of the occurrence of at least one admission; CI: Confidence interval.

The data are expressed as frequency (percentage) for categorical variables, and as mean and standard deviation (SD) and median and interquartile range (IQR) for continuous variables, unless otherwise stated.

^I^ Chi-square or Fisher’s exact tests were used to compare categorical variables and Student’s t-tests or non-parametric Wilcoxon tests to compare continuous variables at each time point.

^II^Admissions to hospital for respiratory and heart-related problems.

^III^ Mean length of stay per admission (hospitalisation), considering only patients who were admitted at least once (12 in the IG and 19 in the CG).

### Impact of home telemonitoring on the length of hospital stay

Considering the patients who completed the 12 month follow-up, the mean length of hospital stay was shorter among patients in the IG for both, all-cause admissions (mean 9, SD 4.3 days vs. 10.7, SD 11.2 days) and cause-specific admissions (mean 9, SD 4.5 days vs. 11.2, SD 11.8 days), although the differences were not statistically significant. A total of 121 hospital admissions were registered among participating patients. Taking into account the causes that led to the hospitalisations, the most common where related to problems with the respiratory system (47.1%), followed by those related to the cardiovascular system (27.3%) and the two conditions occurring simultaneously (10.7%). Overall, 81% of the admissions were cause-specific. The main cause of cardiovascular-related admissions (84.8%) was HF (33.3% in combination with respiratory exacerbation), while most of the respiratory-related admissions (94.3%) were due to acute respiratory exacerbations (37.9% and 19.7% in association with respiratory infections and HF, respectively).

### Effect of home telemonitoring on mortality

At the end of the 12-month follow-up period, 3 individuals died in the IG (12.5%) and as compared to 8 in the CG (26.7%), the difference not being statistically significant (p = 0.310). Among the patients in the IG, the deaths were attributable to HF and COPD relapse in one case, to cholecystitis in another and to multi-organ failure associated with prosthetic valve endocarditis in the last case. Among the patients in the CG, 5 of the deaths were related to problems with the respiratory system, 2 to HF and in 1 case the cause of death was not determined (the patient died at home).

### Effect of home telemonitoring on the use of other healthcare resources

**Figure 2:**
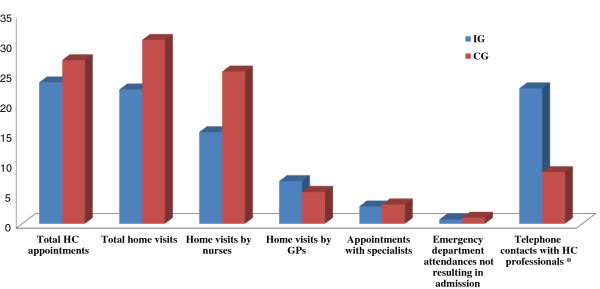
**Comparison of resource use at Health Centres in the IG and CG.** The figures presented were calculated considering the mean per patient among those who completed the 12-months of follow-up. ^*^ The only statistically significant difference was found for telephone contacts (p = 0.001). IG: intervention group; CG: control group; HC: health centre.

**Figure 3:**
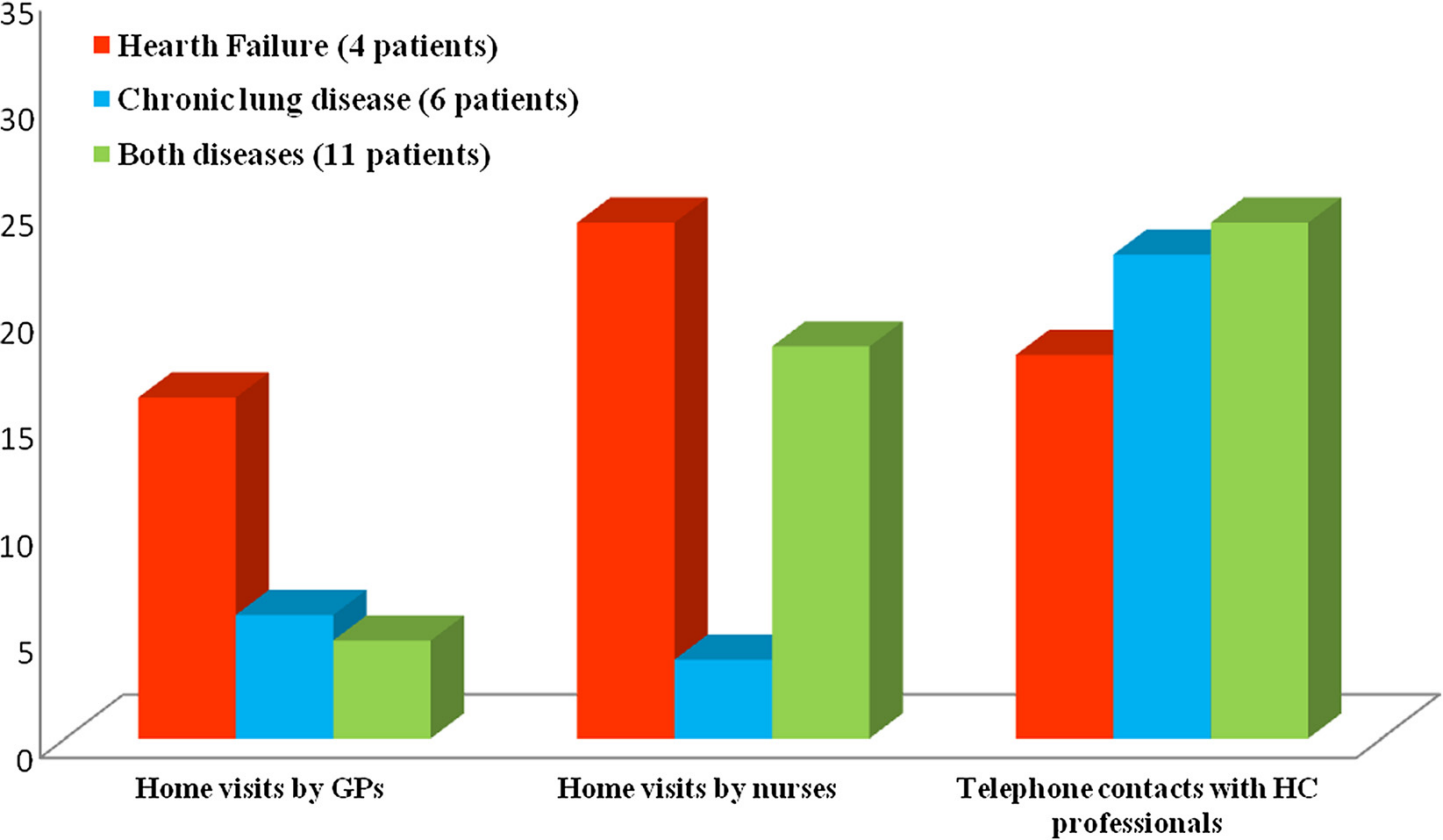
**Comparison of home visits and telephone contacts among patients in the intervention classified by disease.** The figures presented were calculated considering the mean per patient among those patients in the intervention group who completed the 12-months of follow-up.

With regard to home visits, overall there were fewer visits conducted by either GPs or nurses among patients in the IG. Focussing on the analysis of the visits performed by nurses, there were a mean of 15.3 and 25.4 home nursing visits per patient in the intervention and control groups, respectively, though the difference was not statistically significant. The only variable in which significant differences were found between the two study groups at 12 months of follow-up was the number of telephone contacts between the patients and health professionals, this being higher among patients in the IG (p = 0.001) (see Figure 2). Figure 3 shows the comparison of the home visits and telephone contacts registered among patients in the intervention group depending on the inclusion disease (HF, CLD, or both).

The comparison of the hospital admissions and the use of other healthcare resources that occurred in the year prior to the inclusion in the study and the year of follow-up are shown in Table 3. A significant decrease in the number of all-cause hospital admissions in both groups was observed (a mean of 1.3 fewer in the IG and 1.1 fewer in the CG; p = 0.033). We also found a significant increase in the number of telephone contacts in the IG (p < 0.001) in the follow-up period, and a considerable decrease in the number of appointments with the specialists (p = 0.033) and the number of appointments at the primary care health centres (p = 0.015); such changes were statistically different with respect to the changes in the means that occurred among patients in the CG.

**Table 3:** **Difference in healthcare resource use between the year prior to inclusion and the follow-up period**^I^

	**IG**	**p value**^**II**^	**CG**	**p value**^**II**^
	**(n = 21)**		**(n = 22)**	
	**median (IQR)**		**median (IQR)**	
**All-cause hospitalisations**	-2 (-3 to -1)	0.042	-1 (-3 to 0)	0.033
**Cause-specific hospitalisations**	-2 (-2 to -1)	0.086	-0.5 (-1 to 0)	0.244
**Length of stay**^III^	-0.4 (-3.8 to 1.6)	0.733	-1 (-5.5 to 5.3)	0.798
**Health centre appointments**^IV^	-11 (-16 to 0)	0.015	2 (-6 to 6)	0.801
**Total home care visits**	-1 (-2 to 12)	0.734	5 (-4 to 8)	0.152
**Home visits by doctors**	0 (-3 to 3)	1	0 (-2 to 2)	0.745
**Home visits by nurses**	2 (-2 to 4)	0.281	1 (-5 to 9)	0.384
Telephone contacts^*^	10 (6 to 24)	<0.001	3 (0 to 6)	0.147
**Emergency department attendances**	0 (-1 to 0)	0.210	0 (-1 to 1)	0.981
Appointments with specialists^*^	-1 (-2 to 0)	0.033	0 (0 to 1)	0.607

IG: intervention group; CG: control group; IQR: interquartile range.

^I^ A positive/negative difference in the means indicates an increase/decrease in use of the corresponding healthcare resource in the 12-month follow-up period compared to the 12 months before the study.

^II^ Wilcoxon signed-rank test assessing whether the differences between the 12-month prior to inclusion and the 12-month follow-up period were statistically significant, in each group (IG and CG).

^III^ Mean length of stay per admission (hospitalisation), considering only patients who were admitted at least once (12 in the IG and 19 in the CG).

^IV^ Appointments with doctors and/or nurses at the health centre concerning the participating patients, even if the patients themselves were not present.

^*^ Statistically significant differences between the two groups in terms of changes from the year prior to inclusion to the follow-up year: total health centre appointments, p = 0.035; telephone contacts, p < 0.001; and appointments with specialists, p = 0.033. No other statistically significant differences were found.

### Registered telemonitoring alerts

A total of 153.5 (SD 75.8) alerts were generated per patient over the total follow-up period of study for those patients using the telemonitoring devices. The most common cause that triggered such alerts was the detection of clinical values out of the pre-established threshold, specifically for blood pressure (24.7%), respiratory-rate (21.3%) and oxygen saturation (21.2%). The mean number of alerts related to the blood oxygen saturation level and heart-rate in the five days prior to cause-specific admissions was significantly higher than that registered over the entire monitoring period (Table 4). The number of alerts generated by each parameter in the five days prior to the hospital admissions are also shown in Table 4, the highest percentages being found for blood oxygen saturation (74.3%), respiratory-rate (64.9%) and negative responses to the health status questionnaire on the PDA (54.5%). Patients with heart disease had more oedema in their legs (27.3%), followed by nocturia (18.2%), while in the patients with lung disease, alerts were due to increased cough as compared to the previous day (30.3%), increased sputum production and changes in the colour of the sputum (27.3% in both cases). The main measure taken by the health professionals regarding the alerts was registering the alert and taking no further action (82.4% of the cases), followed by making a telephone call (12.6%) or a home visit (3.3%). Thus, in most cases alerts were false alarms.

**Table 4:** **Alerts and mean values in the 5 days prior to cause-specific admissions**^I^_,_**as compared to the entire follow-up period**

	**Value in the entire follow-up period**	**Value in the 5 days prior to hospitalisation**	**p value**^**II**^	**% of alerts generated in the 5-day period prior to hospitalisation**
				**%**
	**mean (SD)**	**mean (SD)**		
**SBP**^III^, mmHg	119.8 (14.7)	121.2 (23)	0.634	38.9%
**DBP**^III^ , mmHg	69.1 (6.7)	70.5 (11.3)	0.640	36.1%
**Blood O**_**2 **_**saturation**,%	93.1 (2.2)	91.0 (4.6)	0.003	74.3%
**Heart-rate**, bpm	77.8 (14.6)	84.2 (17.1)	0.003	27.8%
**Respiratory-rate**, bpm	26.3 (4.3)	26.0 (4.1)	0.703	69.4%
**Body weight**^IV^, kg	74.4 (23.1)	75.5 (23.2)	0.687	31%
**Temperature**, °C	35.9 (0.4)	35.5 (1)	0.059	27.8% ^V^
**Health status questionnaire**^VI^				54.5%

SD: Standard deviation.

^I^ Considering cause-specific admissions to hospital for respiratory and heart-related problems.

^II^ Obtained using the t-test for dependent samples, comparing the overall mean for each parameter and the mean in the 5 days before the admissions.

^III^ SBP and DBP stand for systolic and diastolic blood pressure, respectively.

^IV^ Considering only patients for whom weight data were recorded. Alerts were generated when ≥2 kg were gained over a period of 3 days.

^V^ Only 3 (5.6%) of alerts were for body temperatures over 37°C, the rest being for values below the lower limit.

^VI^ Negative responses to any of the health status questionnaires (on the personal digital assistant) generated alerts.

## Discussion

This study can contribute to shed some light on the impact of home telemonitoring of chronic elderly patients on healthcare resource use. Furthermore, this research provides evidence of the feasibility of the use of information and communication technology (ICT) applications by elderly patients with limited computer literacy. The results of this study show a positive effect of home telemonitoring of in-home patients with a high degree of comorbidity on healthcare resource usage.

The primary outcome measure was the number of hospitalisations at 12 months post-randomisation. In this respect, it was particularly striking that 42.9% of patients (9 people) in the IG completed the follow-up without any hospital admissions (the mean number of admissions in the previous year having been 3.4), and such figure was significantly different from the rate of 13.6% among the patients in the CG. This trend of a decrease in the number of hospital admissions is consistent with the results of other analysis we performed to assess hospitalisations, although in some cases the trend did not reach statistical significance: comparison of the number of admissions per patient; ORs for hospitalisations; NNTs and the comparison of the values in the year prior to inclusion relative to those during the 12 months of follow-up.

The observation that telemonitoring can reduce hospital admission is in agreement with the results of other benchmark studies. In the Whole System Demonstrator study, patients in the IG had fewer admissions, with an OR of 0.82 (95% CI 0.70 to 0.97) [8,9]. Similarly, in the meta-analysis by Polisena and colleagues concerning HF [5] three RCTs [16-18] yielded an overall RR of 0.77 (95% CI 0.65 to 0.90), and in a meta-analysis on COPD by the same authors [6] two studies [19,20] demonstrated a decrease in the number of admissions of 32% to 46% among patients included in the IG. Further, another meta-analysis on HF [21] found a protective effect with a RR of 0.93 for all-cause admissions and of 0.71 for specific admissions due to HF, the latter being statistically significant. The 2010 Cochrane review [4] showed that telemonitoring decreased the number of total hospital admissions and admissions due to HF by 44% and 21%, respectively, thus confirming the findings of earlier research [21].

The majority of the hospital admission that occurred during the completion of the study were cause-specific (81%), that is, for health problems related to one of the two health conditions considered in the present study (HF and CLD). In this respect, 84.8% of the admissions related to cardiovascular problems were due to HF, while 94.3% of respiratory-related admissions were due to respiratory exacerbations. This fact underlines the importance of a good clinical management of the aforementioned conditions and, especially, the significance of the early detection of the episodes of worsening or exacerbation, as the conditions with which patients have been diagnosed are responsible for most of the admissions. In this sense, it is essential that when applying telemonitoring interventions, a good disease management is combined with the deployment of the technology.

Additionally, a trend towards shorter hospital stays among telemonitored patients was also observed. In this regard, the mean days of hospital stay for those patients who completed 12 months of follow-up was of 9 days in the IG vs. 10.7 days in the CG, despite the differences observed were not statistically significant. Moreover, considering the total 121 hospital admissions that occurred during the study, the length of hospital stay due to all causes was 9.6 days vs. 12.2 days and number of days in hospital due to specific causes was 9.8 vs. 12.5, in the intervention and control groups, respectively, although the differences were not statistically significant. Several other authors have reported decreases in the length of hospital stay in patients with HF and COPD [4,6,8,22], but results are not consistent across all studies [23] and some RCTs have shown the opposite effect [22,24].

Regarding the impact of home telecare on mortality, fewer deaths were observed among patients in the IG than in the CG (3 vs. 8 patients) and the mortality-rate was lower than would be expected with respect to estimates based on published data and our statistics from previous years for patients with similar characteristics. Nevertheless, the sample size of the present study is not large enough to draw solid conclusions. To date, several studies have found a lower mortality-rate when telemonitoring was used on HF patients [4,5,8,10,21,23], but there is no consensus for the aforementioned condition [23], and even less consistent results have been published for patients with COPD [6].

During the course of the study, home telemonitoring of the trial patients led to changes in how care was delivered by the participating primary care health centres. In this respect, there were significantly more telephone contacts between telemonitored patients and their GPs and/or nurses (with a mean of 22.6 telephone calls per patient in the IG, vs. 8.5 in the CG) and fewer home visits, mainly due to significantly fewer home nursing visits (with a mean of 15.3 nursing visits per patient in the IG vs. 25.4 in the CG). A very slight increase in home visits by GPs was however observed in the IG, which we believe may be influenced by a closer delivery of care and increased initial actions undertaken by doctors, until the participating GPs became familiar with the telemonitoring process. Thus, assuming that the intervention is effective decreasing the number of hospitalisations, such redistribution of healthcare activities and resources may entail savings, considering the costs associated with telephone contacts compared to home visits. On the other hand, these changes could have a significant impact on the way primary care professionals work, leaving more time for nurses to spend on other relevant task that could improve the management of this type of patients. Nonetheless, the impact of such operational modifications has not been directly addressed in this study and requires further investigation. Few studies have assessed the effect of home telemonitoring on telephone calls and home visits and although some studies have been consistent with our findings [20], others were not [4].

We believe that when assessing the impact and overall implications of home telemonitoring it is essential to consider the global effects of the technology itself in addition to the usage of healthcare resources. Thus, other complex factors such as, the organizational changes, standards and perceptions of health and safety, effect on the patients’ QoL, economical implications, patients’ and healthcare professionals’ satisfaction as well as the effect of telemonitoring on family caregivers should also be taken into account. Some of these aspects have been assessed by our team and will be published shortly. A cost-effectiveness analysis of the TELBIL study has also been undertaken [25].

We would like to highlight three key characteristics that differentiate this study from others and make it particularly relevant. Firstly, the telemonitoring was managed by primary care professionals (GPs and nurses) who regularly see the patients in the health centres or at home. The fact that primary care professionals are in charge of the telemonitoring intervention is particularly important, since these are the healthcare professionals that routinely carry out the follow-up of in-home patients and, thus, telemonitoring could have a greater positive impact than when applied to hospital-based interventions. In this regard, the integration of the new telemonitoring intervention to the routine practice at the health centres could improve the care provided. To the best of our knowledge, this is one of the first RCTs in which primary care professionals are in charge of the telemonitoring procedure. The implication of primary healthcare professionals has been further explored in the present study. On the one hand, the implication of around 70 primary care professionals has enabled us to undertake a qualitative analysis with focus groups to further assess the satisfaction and specific contribution of the participating GPs and nurses [26]. On the other hand, the factors related to the healthcare professionals’ acceptance of the new telemonitoring technology have been evaluated through and extension of the Technology Acceptance Model (TAM), showing that the perception of facilitators in the organisational context is the most important variable to consider for increasing healthcare professionals’ intention to use the telemonitoring technology [27].

Secondly, the patients included in the present study had challenging characteristics: they had a higher mean age (81 years) than that targeted by most other published studies [4-6] and were patients with advanced diseases and high levels of comorbidity (51.7% were under home oxygen therapy, 46.5% had both of the diseases targeted in this study, 86.2% scored high comorbidity (>2) on the Charlson index and 79.3% showed signs of clinical deterioration) and, in line with these features, the patients included in the trial were heavy users of healthcare resources. In the view of the above mentioned peculiarities, this trial demonstrates the feasibility of implementing telemonitoring interventions as an alternative mode of health care provision for medically unstable patients with high degree of physical and functional deterioration. Furthermore, there were only 5 individuals not wishing to continue using the telemonitoring system and even these had all successfully managed to handle the devices. The observed low dropout-rate contrasts with other research into the feasibility and perceptions of this technology, which suggests that the older the patient and/or caregiver the more obstacles to the adoption of telemonitoring [28].

Thirdly, we have focussed on two common chronic disorders (HF and CLD), which have been managed using the same telemonitoring system and in a very similar way, while practically all previous RCTs on telemonitoring technology have targeted a single chronic condition [29]. This point is important, since the levels of comorbidity among this kind of patients make it difficult to consider a single condition in isolation from other existing health problems. Hence, the telemonitoring management approach should be adapted to reflect the clinical and functional status of the patients, rather than focussed on a specific disease.

### Study limitations

The results of our study should be interpreted in light of some limitations. First, the number of patients included in the study was limited by the available telemonitoring devices. Twenty eight patients received telemonitoring and 21 patients in the IG and 22 patients in the CG completed the follow-up. We have, therefore, observed a deviation in the estimated power mainly due to an increase in the expected losses and a lower reduction in the number of hospital admissions than previously envisaged. We have recalculated the statistical power, taking into account the results obtained, with a confidence level of 95% in a bilateral contrast and 21 patients in the IG and 22 in the CG. Thus, the present study has a statistical power of 58% to detect significant differences between the percentages of patients with ≥1 hospital admissions in the IG (57.1%) and in the CG (86.4%).

Another limitation of this study is that due to the interactive nature of the intervention, it was not possible to blind the health care professionals providing the intervention or the participants involved in the study. However, despite this limitation, most of the data presented in this manuscript are objective and have been obtained from medical registers. The veracity of the data obtained has been double-checked and the statisticians in charge of the data analysis have been blinded to group assignment.

## Conclusions

Our study shows that primary care-based telemonitoring increases the percentage of in-home patients with no hospital admissions after 12 months of follow-up. We also observed trends towards fewer all-cause and cause-specific admissions, as well as shorter hospital stays.

We have also observed that telemonitoring leads to a significant increase in the number of telephone contacts between healthcare professionals and patients, but this is balanced by a decrease in the use of other healthcare services provided by primary care health centres, particularly, home nursing visits.

The present study demonstrates the feasibility of this mode of healthcare provision in elderly patients with high levels of comorbidity and limited computed literacy.

Prior to the implementation of telemonitoring interventions, it is essential to reach beyond the mere technological aspects, paying attention to the proper clinical management of the patient’s condition and the impact of the telemonitoring on healthcare professionals, patients and their families.

Further studies are required in primary care settings involving patients with common chronic illnesses and comorbidities. Future research should also analyse the different elements of the overall intervention, identifying those with the greatest effects.

## Competing interests

The authors declare that they have no competing interests.

## Authors’ contributions

IML, as the principal investigator, participated in all aspects of the project, coordinating the different stages of the study. EO has conducted the critical revision of the manuscript and has participated in the conception and design of the study and the interpretation of the study results. AB has conducted the statistical analysis and has participated in the interpretation of the study results. IV has contributed to the design of the study and interpretation of the study results. MCC and RA have principally contributed to the study design and the assessment of patients. JCB has contributed to the drafting of the economic analysis and has participated in the design of the study and interpretation of the results. ER has helped with the evaluation of the satisfaction of patients and health care professionals with the telemonitoring technology. JA, MIR and JL have been in charge of institutional relations, helping with the dissemination of the results and liaising with the company that supplied the technology. All authors have read and approved the final version of the manuscript.

## Pre-publication history

The pre-publication history for this paper can be accessed here:

http://www.biomedcentral.com/1472-6963/13/118/prepub
